# Profile of judicialization in access to antineoplastic drugs and their costs: a cross-sectional, descriptive study based on a set of all lawsuits filed between 2016 and 2018 in a state in the Northeast Region of Brazil

**DOI:** 10.1186/s12889-022-14199-1

**Published:** 2022-09-26

**Authors:** Fábio Henrique Cavalcanti de Oliveira, José Eudes de Lorena Sobrinho, Antônio da Cruz Gouveia Mendes, Haynne Magalhães Santos Gutman, Geraldo Jorge Filho, Ulisses Ramos Montarroyos

**Affiliations:** 1 Medical School, University of Pernambuco, Recife, PE Brazil; 2 Institute Aggeu Magalhães (IAM-Fiocruz), Recife, PE Brazil; 3 Pernambuco Health’s Secretary, Recife, PE Brazil; 4 Institute of Biological Sciences, University of Pernambuco, Recife, PE Brazil

**Keywords:** Judicialization of healthcare, Pharmaceutical assistance, Antineoplastic drugs, Drug costs, Costs and cost analysis

## Abstract

**Background:**

The judicialization of the acquisition of medication for healthcare is not restricted to Brazil but can also be found in other Latin American countries, despite the existence of a universal health system in the case of Brazil, the Unified Health System (known as the SUS). Right-to-medicines litigation has existed ever since the emergence of a high demand for treatment of Acquired Immuno-deficiency Syndrome (AIDS) but the current focus is on cancer. Pharmaceutical Assistance (PA) is the area within the SUS that is responsible for ensuring access to medication and the aim of this article is thus to draw up a profile of litigation related to PA in one economically significant state in the Northeast Region of Brazil, in terms of the following characteristics of lawsuits: the plaintiff filing the lawsuit; medical and health information; the cost of acquiring the requested medications; and the proportion accounted for by spending on antineoplastic drugs.

**Methods:**

A cross-sectional, descriptive study was conducted of lawsuits filed between 2016 and 2018 at the Litigation Center of the State of Pernambuco Department of Health.

**Results:**

A total of 2,947 lawsuits containing at least one requested medication were analyzed. The majority of the plaintiffs were male (51.7%); 49.8% of the requests originated in the Unified Health System (SUS), and plaintiffs were primarily patients in the Metropolitan region of the State capital, Recife. The most frequent cancers involved were those classified by the ICD as C61, C71 and C50. The median general expense on medications for the actions was U$1,734.94. Considering antineoplastic drugs alone, the cost exceeded U$7,500 per lawsuit over the three years, given that the median unit price for antineoplastic drugs is approximately US$65 compared to US$4 for non-antineoplastic drugs.

**Conclusion:**

The present study is of relevance to the field of public health and examines how a profile of such healthcare litigation can be used as a tool for managing and improving decision-making in times of economic austerity.

**Supplementary Information:**

The online version contains supplementary material available at 10.1186/s12889-022-14199-1.

## Introduction

The judicialization of healthcare involves health-related goods and services being acquired by way of litigation [1], by groups or individuals, to meet health needs (access to services and supplies) that are not being met by the executive branch of government [2]. As the State is duty bound to uphold the basic rights of all citizens in an equitable manner [3], the judicialization of the right to healthcare has divided scholarly opinion in the field [4].

Access to medication in Brazil is provided by the SUS—National Health System, with the possibility of recourse to litigation on occasions when a citizen is denied this right by the health system [5]. Since the 2000s, judicialization has become a major issue in Brazil as a result of the demand for new drugs to treat Acquired Immuno-deficiency Syndrome (AIDS). During this period, 92 such drugs have been launched [1, 4]. At present, anticancer drugs are the fastest growing category. The incidence of cancer is increasing globally and this is putting significant pressure on populations [5] at all levels of income and on all health systems. This has given rise to international action to promote increased commitment to greater investment in disease control as a public health priority [6]. Irrespective of the reasons for the increase in cancer detection rates, the need to treat and cure this disease has generated greater demand for medication and caused the lawsuits relating to access to high-cost antineoplastic drugs to feature more prominently in the judiciary system [2, 5].

It should be noted this increase in right-to-medicines litigation is not restricted to Brazil but can also be observed in other Latin American countries [2]. This increase has occurred even in cases where a universal health system or even a universal right to health is guaranteed by the country’s constitution, as is the case in Peru, Argentina, Venezuela and Ecuador [7].

The SUS department responsible for managing medication is called the Department of Pharmaceutical Assistance (PA) and this department is responsible for actions that aim to promote, protect and recover health, both at individual and collective level, in cases where medication is essential [7]. It is for this reason that the PA is the main focus of the debate regarding litigation [8]. Growing judicialization [4, 9] suggests that there is a need for careful analysis of policies and programs relating to provision of access medication, and for examination of the lists of essential medicines furnished by Pharmaceutical Assistance, as a way of ensuring efficient and adequate healthcare outcomes [10].

State Health Departments have recently been developing strategies to resolve such legal issues and reduce the impact of such lawsuits on various spheres of public administration in Brazil, principally healthcare. Nantes and Dobashi [11] cite the creation of a “coordinator of legal action”, linked to the State’s management board, which comprises a multidisciplinary team working in collaboration with the State Prosecutor on various issues, including lawsuits relating to the right to medicines [12, 13].

Even with this restructuring of State Health Departments, the judicialization of healthcare is still growing tendency, as litigation is an effective alternative means of acquiring a product, service or treatment related to health that is not covered by existing health system protocols. In the case of medication, basic protocols are treated separately and, even so, some medications are unavailable, owing to a lack of the requisite technology or insufficient funding [4, 14].

In 2016, in the State of Pernambuco, lawsuits relating to the purchase of medication were the most prevalent, accounting for around 63.5% of the lawsuits filed against the State Department of Health [14]. In a final ruling of the Federal Court of Accounts—TCU (2017) [15], most Ministry of Health spending on court cases relating to medication concerns items that have not been incorporated into SUS protocols.

In the State of Pernambuco, an agreement has been established (048/2011) [16] between the State Health Department, the Court of Justice and the Legal Action Unit, at which court cases are dealt with by a Technical Advisory Unit for Health. This is used as a management tool to ensure that the public demand for health services is met and to reduce the number of court cases, as recommended by the Brazilian National Justice Council (CNJ) [12].

As described in a study by Nunes and Ramos Júnior [13], there are no clear data or no data at all on the extent to which healthcare has become judicialized in Brazil, nor on temporal or geographical distribution of this trend. Data for specific regions of the country are inconsistent or non-existent, especially so far as the demand for antineoplastic medication is concerned.

The present study aims to present a profile of the judicialization of medication in the State of Pernambuco, according to the characteristics of the lawsuits [17], including: the plaintiff filing the lawsuit; medical and health information, the cost of acquiring the required medications; and the proportion of this expense accounted for by antineoplastic drugs.

## Method

A cross-sectional, descriptive study was conducted of all lawsuits filed between the years 2016 and 2018 at the Litigation Center (NAJ) in the State of Pernambuco’s Department of Health (SES/PE).

Data were initially collected by a trained, qualified team from the Litigation Center, using internal forms that contained detailed information on each lawsuit involving the State as a defendant.

An adapted version of the original proposal of Pepe et al. [17–19] for selection of variables of interest was employed, including variables related to the characteristics of the plaintiff (sex and municipality of residence), medical and healthcare related characteristics of the lawsuits (origin of prescription of medication according to legal status with regard to the National Register of Health Establishments—CNES); principal diagnosis, according to the International Classification of Diseases – 10^th^ Revision (ICD 10); and the cost of acquiring the requested medication.

Lawsuits were included if they had been filed against the State of Pernambuco and received at the Litigation Center, if they mentioned at least one drug, and if it was possible to establish the price of the suggested dose during the study period. Lawsuits were excluded if they contained insufficient information or involved drug combinations or formulations in which individual drugs could not be priced separately.

In the case of medication with more than one treatment indication, we classified it according to the first prescription suggestion given in the Anatomical Therapeutic Chemical Classification System (ATC code).

Spending on litigation relating to medication during the period 2016–2018 was estimated using the quantity requested for purchase during the period, according to the Pharmaceutical Assistance Management System (SISGAF) of the Litigation Center, and the acquisition price given in the Minutes of the SES/PE Price Registration. For the purposes of comparison, values given in Brazilian reais have been converted into dollars using data provided by the IPEA—Institute of Applied Economic Research, (R$)/US dollar (US$) based on a mean exchange rate for 2020 of R$5.1558 to the dollar.

For unidentifiable costs, the Healthcare Prices Bank (BPS) was used as a parameter. For the study period, all prices were consulted in the BPS dated December 2019, or, failing this, by consulting the factory price suggested on the distributors' website. This methodology was used to minimize the effect of possible price variations during the consultation period.

Data analysis compared spending by the various characteristics of the lawsuits and type of medication (non-antineoplastic and antineoplastic), using the Kruskal–Wallis test for comparison of medians, and Pearson's Chi-square test to compare proportions. The Shapiro-Wilks test was used to confirm the hypothesis of normality of the variable related to expense and the hypothesis of normality was rejected. The statistical significance adopted was 5% (*p* < 0.05). Data were analyzed using Stata 14.

Once the values for each variable of interest had been identified, indicators were calculated for each municipality in which the lawsuits originated. These indicators were used to draw up quantitative thematic maps. A digital map in the shapefile format, as provided by the Brazilian Institute of Geography and Statistics (IBGE), was used and the calculated rates were added to this. The software used was QGIS 3.16.10 and the classes used for the quantitative thematic maps were divided into quartiles.

The study was approved by the Research Ethics Committee (CEP) of the University of Pernambuco (UPE), on July 3, 2018, under **CAAE**: 91652318.3.0000.5207. As the study did not directly involve human beings, but used secondary data, the aforementioned Committee decided to approve the project, exempting it from the requirement to use an informed consent form. All methods respected ethical guidelines and regulations.

## Results

Analysis was conducted of 2,947 lawsuits involving at least one request for medication filed against the State between the years 2016 and 2018, the characteristics of which are presented in Table 1.

**Table 1:** Characteristics of lawsuits filed against the State of Pernambuco and medications involved, 2016 and 2018

Characteristics	n (%)
**Number of lawsuits**	2,947
**Sex**	
Male	1,522 (51.7%)
Female	1,425 (48.3%)
**Origin**	
Private	667 (23.4%)
SUS	1,419 (49.8%)
Philanthropic	762 (26.8%)
Not available	97
**Number of medications**	
One	2,903 (98.5%)
Two	33 (1.1%)
Three or more	11 (0.4%)
**Antineoplastic Drugs**	
Yes	1,182 (41.4%)
No	1,671 (58.6%)
Not available	94

Most cases involved male patients (51.7%) and the highest number of requests came from the SUS (49.8%), followed by philanthropic hospitals (26.8%). Most lawsuits involved one requested medication (98.5%) and 41.4% of the medications were antineoplastic.

Table 2 shows the expense, in dollars, on lawsuits filed. The median overall expense on lawsuits (regardless of type of drug), in dollars, was US$1,734.94. However, expense on antineoplastic drugs alone exceeded US$7,500 per lawsuit, given that the median value for the unit price of antineoplastic drugs was approximately $65 and the median for non-antineoplastic drugs was approximately $4.

**Table 2:** Cost of medicine, in dollars, on lawsuits involving medication in the period between 2016 and 2018

**Characteristics**	**General** (n = 2,947)	**Antineoplastic Drugs** (*n *= 1,182)	**Non-Antineoplastic Drugs** (*n* = 1,671	***P*-value**
Total expense on lawsuit	1,734.94 (232 – 7,792)	7,508.83 (2,402 – 15,571)	585.36 (86 – 2,635)	< 0.001
Unit price of medication				
In dollars	17.99 (1.94 – 142.23)	64.91 (14.60 – 359.40)	4.09 (0.53 – 65.87)	< 0.001
Sex	p-value^a^ = 0.019	*p*-value^a^ = 0.131	*p*-value^a^ = 0.007	
Male	2,120.33 (245.16 – 8,761.78)	8,654.14 (2,875,.79 –15,578.96)	468.40 (54,11 – 2,546.65)	< 0.001
Female	1,405.02 (220.92 – 6,621.09)	6,492.30 (1,928.70 –15,410.61)	618.53 (115.21 – 2,956.67)	< 0.001
Origin	*p*-value^a^ < 0.001	p-value^a^ = 0.071	p-value^a^ = 0.251	
Private	993.83 (168.74 – 4,948.80)	7,785.41 (3,244.89 –15,949.03)	595.06 (116.96 – 1,646.50)	< 0.001
SUS	2,482.45 (224.41 – 9,374.88)	7,964.82 (2,875.79 –15,410.61)	589.43 (81.66 – 3,556.58)	< 0.001
Philanthropic	2,128.67 (315.25 – 8,633.58)	6,475.23 (1,752.20 –14,170.84)	448.04 (52.37 – 2,634.51)	< 0.001
Number of medications				
One	2,903 (98.5%)	1,255 (99.7%)	1,719 (97.8%)	< 0.001
Two	33 (1.1%)	0 (0%)	31 (1.8%)	
Three or more	11 (0.4%)	4 (0.3%)	7 (0.4%)	
Maximum duration of treatment				
Months	6 (3 – 9)	5 (3 – 9)	6 (3 – 9)	< 0.001

N.B.: Sums in Brazilian reais were converted into US dollars using the commercial exchange rate for selling: real (R$)/US dollar (US$), mean value for 2020 (R$5,1558). Source: IPEA Retrieved from http://www.ipeadata.gov.br/ExibeSerie.aspx?serid=31924 August 2021

The difference in expense as a whole was found to be statistically significant when comparing the medians by sex. Males presented a higher median cost but there was no significant difference in the case of antineoplastic drugs (*p* = 0.131). There was a statistical significance when expense on non-antineoplastic drugs was compared by sex, the higher expense being among females.

There was a statistically significant difference in overall expense on all medications between different types of institutions filing a lawsuit, with higher expense on lawsuits filed by the SUS (U$2,482.45) and by philanthropic entities (U$2,128.67), compared to private entities.

For the most part (98.5%), the lawsuits studied in this period involved just one medication, with a median duration of treatment of six months, and slightly shorter duration of five months for antineoplastic drugs.

The five most frequent ICD diseases involved in these lawsuits were: C61-Malignant neoplasm of the prostate (9.0%); C71-Malignant neoplasm of the cerebral ventricle (4.2%); C50-Malignant neoplasm of the breast (3.8%); N18.8-Other chronic kidney failure (3.7%); and C64-Malignant neoplasm of the kidney (3.5%).

Of the 15 most commonly requested drugs, the ones most frequently requested were Abiraterone, Cinacalcet, Somatropin 12UI, Sorafenib, Bevacizumab, and Enzalutamide. Cinacalcet and Somatropin 12UI are not used for neoplasms.

**Fig. 1:**
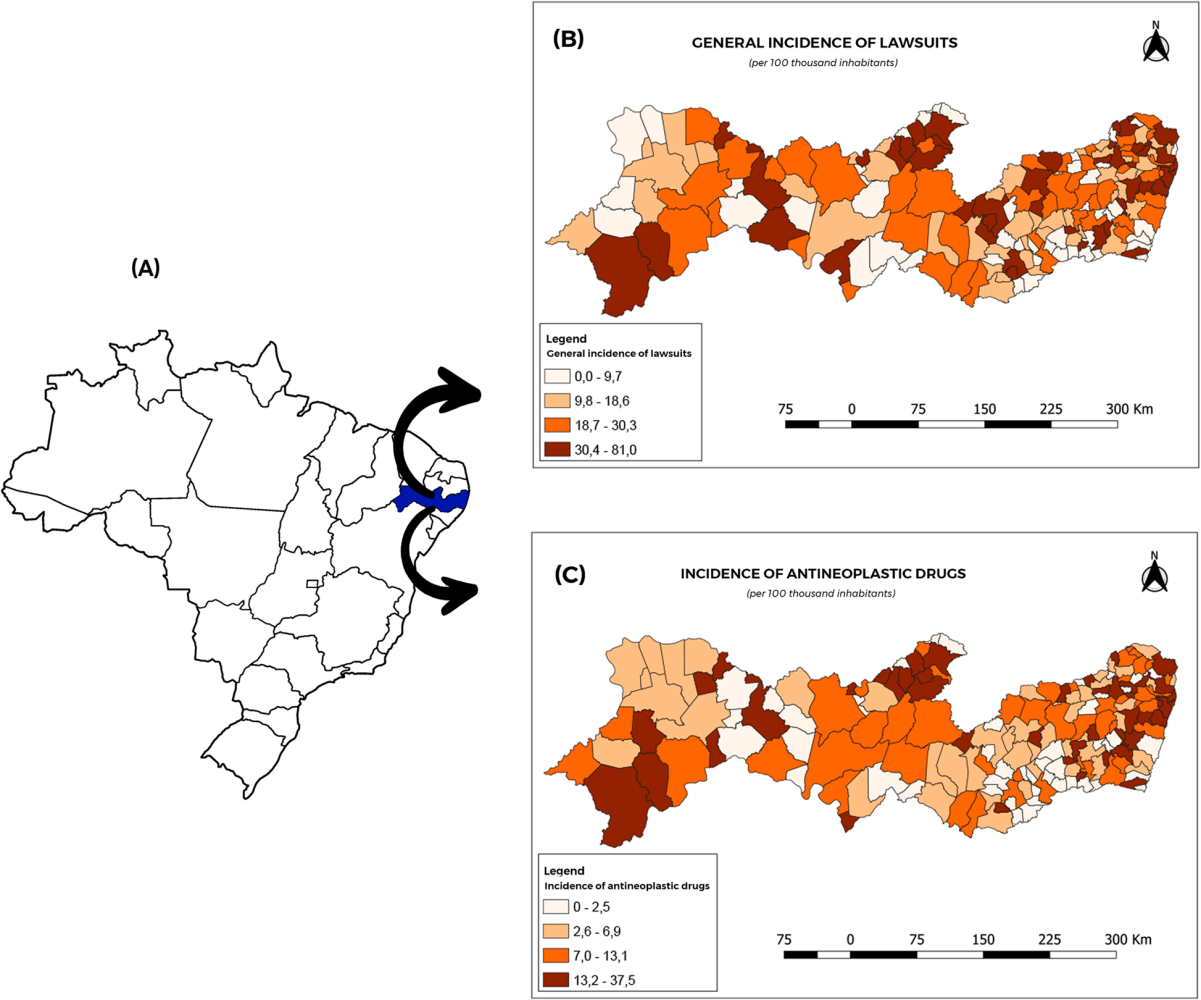
Overall incidence of lawsuits involving general medication (**B**) and involving antineoplastic drugs (**C**) filed against the State of Pernambuco for any medication, per 100,000 inhabitants by municipality, for the period from 2016 to 2018

Analysis of the geographic distribution of the lawsuits with regard to the municipality of origin, revealed, as shown in Fig. 1, the overall profile of incidence of lawsuits per thousand inhabitants, with a higher prevalence in municipalities of significant economic importance for the State, in the metropolitan region, near the State capital, or in the western portion of the State.

During the period studied, the median total expense on litigation in the 184 municipalities was US$20,500 (6,700 – 92.500), with a minimum of US$500 and a maximum of US$7,600 (State Capital). A total of 171 municipalities filed at least one lawsuit for medication (171/184 = 93%) against the State of Pernambuco. The median number of lawsuits for the 184 municipalities during the three-year period was five (2—11), although, in the state capital, there were 876 lawsuits, representing an average of 292 lawsuits per year.

The median number of lawsuits for antineoplastic drugs by municipality over a three-year period was 2 (1—4.5), with a maximum of 388 lawsuits in the State capital. One hundred and forty municipalities filed at least one lawsuit (76%) against the State of Pernambuco.

**Fig. 2:**
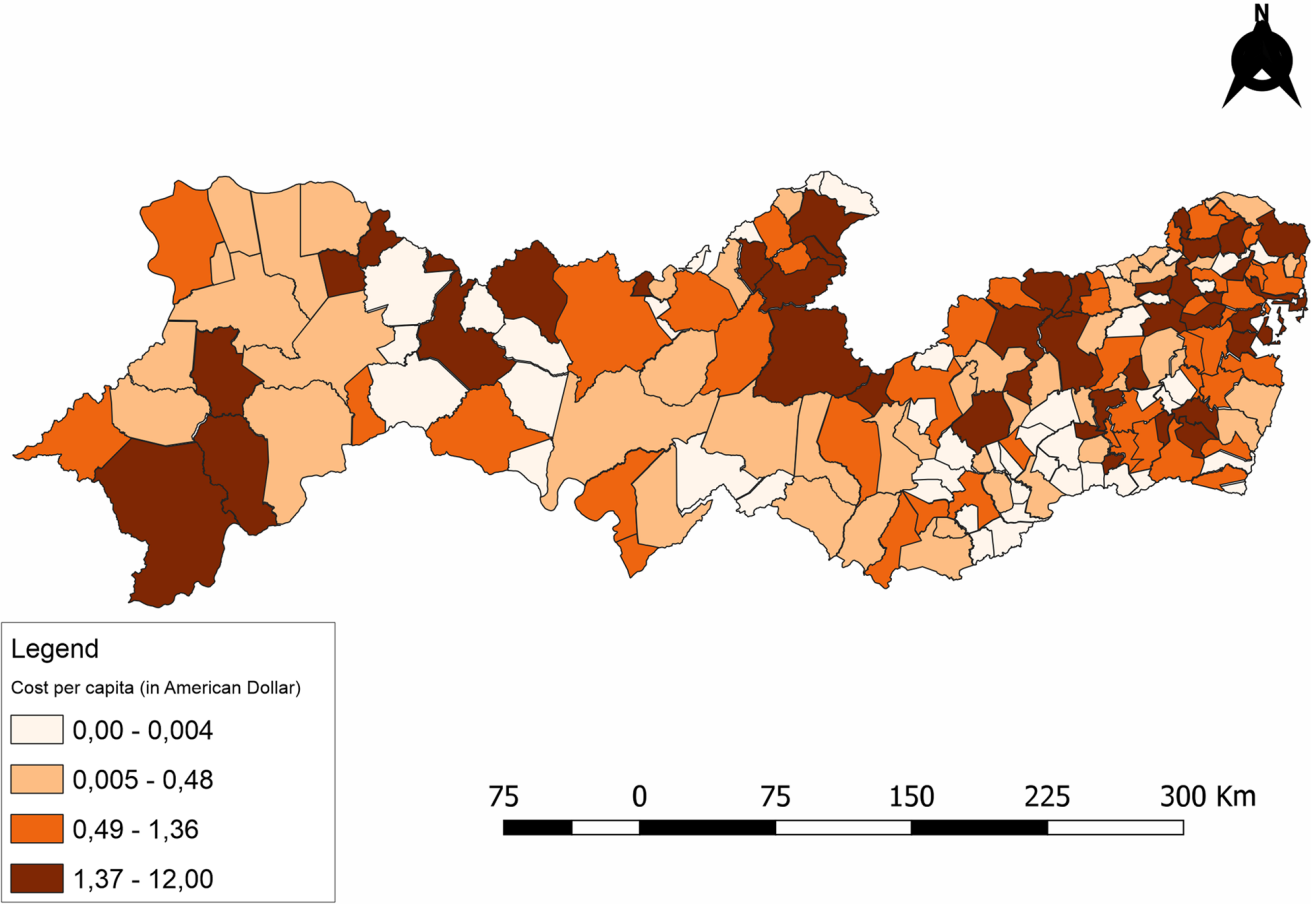
Per capita cost of lawsuits filed against the State of Pernambuco for antineoplastic drugs, between 2016 and 2018

Analysis of the distribution of lawsuits by municipality in Pernambuco (Fig. 2) shows that the median per capita expense during the period for antineoplastic drugs was US$0.8 per inhabitant (0.4—1.9), with the two highest being US$ 12 per inhabitant (Jaqueira) and US$ 2.9 in Recife.

## Discussion

Despite its complexity, it is worthwhile discussing the issue of the judicialization of healthcare in Brazil, especially in so far as this concerns access to medication. This is especially important, since, after decades of studies, no national profile has yet been produced. Likewise, there is no consensus in the national and international literature regarding the demographic profile of those who take legal action to ensure that they receive services and products related to health. The socio-economic status of these individuals remains unclear. There is also no consensus among scholars as to whether these litigants are demanding access to drugs and services that are already included in lists, protocols and contracts or some other kind of drugs and services. Similarly, there are differences of opinion regarding the extent of the disruption that such lawsuits cause to public and private healthcare in general. These questions were raised by the most recent study conducted by the National Justice Council – CNJ, in 2019, [20] but answers are still lacking, owing to the extreme economic inequality between regions in Brazil. This article presents, describes and discusses the profile of lawsuits related to access to medication, especially antineoplastic drugs, in one State in the Northeast region of Brazil, which is an economically disadvantaged region with marked inequalities. It is essential, however, that studies of this nature be produced on a regular basis.

To fill a gap in the data for the Northeast Region of Brazil, the present study examined lawsuits filed against the State of Pernambuco. As this was a cross-sectional study, it was not possible to address any trends that may have increased or reduced the impact of these lawsuits on healthcare management in the State. It was possible, however, to describe the characteristics and trends relating to expense on medication and the proportion of this associated with antineoplastic drugs in a Northeastern Brazilian state that is one of the wealthiest (in terms of GDP) in the region. Attention should be drawn to a few shortcomings regarding the following comparison of our findings with those of other studies in the literature, in view of the variety of methodologies that have been used to collect and describe the characteristics of lawsuits, as recently noted by Oliveira et al. (2021) [21].

Our description of the profile of litigation relating to healthcare in the State of Pernambuco found that the proportion of lawsuits was similar for both sexes, thereby corroborating the findings of Diniz et al. [22], who found that 51% of such lawsuits in the Federal District (DF) were filed by men. Other studies found this proportion to be 52.5% in the State of Ceará [13] and 52.4% in one municipality in the State of São Paulo (SP) [23]. Another study, however, found that 60.2% of lawsuits were filed by women in the State of Minas Gerais [24].

In relation to the type of organization filing the lawsuit, we found that organizations belonging to the SUS were the most common, in accordance with the findings of other studies, in the State of São Paulo (48%) [25], the Federal District [22] (85%), and the State of Ceará [13] (76.3%). It should be noted, however, that the studies conducted in the Federal District and Ceará did not distinguish public and philanthropic institutions. Other findings, however, do not accord with those of the present study. These include the finding that most lawsuits in the State of Minas Gerais originated in the private sector (70.5%) [24]. In short, the profile of healthcare-related lawsuits in the State of Pernambuco is peculiar to this State.

A study by Machado et al. [24] identified that 66.3% of lawsuits requested only one medication, while, in the present study, this figure was 98.5%, corroborating the finding that multiple pharmacotherapeutic drugs tend not to be included in the same lawsuit.

With regard to the low demand identified for antineoplastic drugs, this was not a characteristic peculiar to this study, since, according to the main ATC classification, antineoplastic agents and immunomodulators are one of the main groups requested by way of legal action. They are, however, usually not the most prevalent, as noted by Chieffi and Barata [25], who identified only 33% of lawsuits involving antineoplastic drugs. Such drugs are, however, generally the most expensive.

As median public expense per capita on healthcare in Brazil stands at around R$1,400 per year [26] (approximately US$271.53), it is clear that these lawsuits relating to antineoplastic drugs (costing on average US$7,508.8) may have a significant impact on the public health budget.

Detailed values make it possible to identify the unit price of medication, bearing in mind that price and choice of treatment are the main factors involved in increasing spending on medication. Inference along these lines is widely discussed by Vieira (2019) [27], who clearly outlines the causes underlying public expense on medication, identifying the three main causal factors as being cost, quantity and treatment choice, with variations in quantity and choice of treatment being particularly significant factors in generating increased expense on medication.

An examination of the drugs most frequently involved in lawsuits reveals that Cinacalcet and Somatropin 12UI are not primarily prescribed for neoplasms, even though there has been a history of Cinacalcet being prescribed for the treatment of parathyroid carcinoma and primary hyperparathyroidism. These two drugs are included in the list of 15 drugs most frequently involved in lawsuits. In the case of Abiraterone, Sorafenib and Bevacizumab, which are drugs indicated for oncology treatment, findings are similar to those of other studies, such as Vidal et al. (2017) [2], in INCA-RJ and Barreto et al. (2019) [19] in SES-PE.

Following numerous studies and requests, Abiraterone was, in 2019, incorporated into SUS drugs protocols by the Ministry of Health through the Ordinance of the Department of Science, Technology, Innovation and Strategic Health—SCTIE No. 38/2019 [28]. In 2015, Cinacalcet, which is recommended for conditions ranging from secondary hyperparathyroidism (HPTS) to chronic kidney disease, was incorporated into SUS protocols by SCTIE/MS Ordinance No. 48/201529 [29]. This drug is also indicated for parathyroid carcinoma and primary hyperparathyroidism but is not yet recommended by the SUS for this purpose. For this reason, the use of off-label medications is a constant subject of debate, not only in the SUS but also in the National Health Agency (ANS), which is responsible for regulation of the supplementary system [2].

An analysis of the lawsuits by municipality enables a direct correlation to be drawn in relation to the municipality and the socioeconomic and health management region of the state, since Recife (29.4%), Jaboatão dos Guararapes (7.4%), Olinda (5.3%) and Paulista (4.3%) are located in the Metropolitan Region of Recife and Health Region I, and together account for approximately 57.1% of the lawsuits. Studies such as those of Nunes and Ramos Júnior [13] have reported similar findings, observing that, in more than 77% of lawsuits, the municipality of residence of the plaintiff was Fortaleza, the capital city of the State of Ceará.

The data on the municipality of residence of plaintiffs confirm the profile of the State but it cannot be argued that the socioeconomic profile of the plaintiffs influenced the profile of judicialization in Pernambuco, nor was this the aim of the present study.

Figueiredo et al. [30], in a national study using data for 2014, estimated total spending per capita on healthcare in Brazil to be US$ 947. If we consider only one disorder, such as cancer, the expense is US$12, as found in the present study. This significant finding suggests that more detailed studies should be dedicated to the highly important issue of which types of disease account for the greater share of healthcare spending. Figueiredo et al. [30] claim that Brazil spent a smaller quantity of public funding daily per capita on public health services and related actions (R$ 2.60—US$ 0.50) compared to other countries that possess universal healthcare systems. There would appear therefore to be a discrepancy between expense on lawsuits relating to anticancer medication in Pernambuco compared to overall daily public spending per capita on public health services and related actions.

Finally, the 2,947 medication lawsuits reported in this study have made it possible to describe and discuss the profile of healthcare litigation in one State in the Northeast of Brazil, including financial aspects of relevance for future discussions regarding the financial impact on public health management.

## Final considerations

From a descriptive analysis of a state in northeastern Brazil, a profile of judicialization equally demanded by gender, half of them originating from demands from the public health system, with an average cost of antineoplastic drugs, approximately, 13 times higher when compared to lawsuits involving other drugs. According to the distribution of cases among the municipalities in the state of Pernambuco, those located in the metropolitan region concentrated a greater number of lawsuits.

It is evident that there is a need to provide more extensive information on the growing use of the legal system to acquire access to medication. Further investigation of requests for antineoplastic drugs and their concentration in the Metropolitan Region of Recife, the state capital is nevertheless required as a way of ascertaining whether such lawsuits genuinely help to uphold the rights of all citizens, regardless of socioeconomic status.

Further research needs to be conducted in this field, since the various studies already published have for long indicated that judicialization forms part of the concrete reality that healthcare managers are obliged to deal with, irrespective of the legal, economic or even social perception that individual rights are being upheld to the detriment of collective rights. This reality may or may not, of itself, help to bring about changes in healthcare policy and should be seen, therefore, as an ongoing process of developing and evaluating adjustments to the healthcare management model and ensuring that future healthcare policies are more closely aligned with technological advances in the development of medication for cancer and other diseases.

Furthermore, while the funding for a health system first envisaged in the 1998 Constitution is clearly now insufficient, the situation has been further exacerbated by the fiscal austerity policies recently introduced in Brazil. This is of great significance for the subject under discussion here, since it is always possible to determine the financial impact of judicialization in objective terms. It should therefore be possible to use this objective data to shed greater light on expense and thereby help to validate new public policies that aim to reinforce efforts to ensure a truly universal and public health system.

## Supplementary Information

**Additional file 1.** Database.

## Data Availability

The data that support the findings of this study are available from SES: State Health Departments but restrictions apply to the availability of these data, which were used under license for the current study. The autors processed the data, hiding the identity of the litigants. The data use in the study is publically, as it is a public expense.

## Abbreviations

AIDS: Acquired Immunodeficiency Syndrome
CEP: Research Ethics Committee
CNES: National Register of Health Establishments
CNJ: National Council of Justice
DF: Brazilian Federal District
HPTS: Secondary Hyperparathyroidism
IBGE: Brazilian Institute of Geography and Statistics
NAJ: Litigation Center
NATS: Center for Technical Assistance in Health
PA: Pharmaceutical Assistance
RENAME: National List of Essential Medicines
SES-PE: Pernambuco State Health Department
SES: State Health Departments
SISGAF: Pharmaceutical Assistance Management System
SP: São Paulo
TCU: Federal Court of Accounts
TJPE: Pernambuco Court of Justice
UPE: Universidade de Pernambuco
SUS: Unified Health System
